# Prediction of RNA secondary structure with pseudoknots using integer programming

**DOI:** 10.1186/1471-2105-10-S1-S38

**Published:** 2009-01-30

**Authors:** Unyanee Poolsap, Yuki Kato, Tatsuya Akutsu

**Affiliations:** 1 Bioinformatics Center, Institute for Chemical Research, Kyoto University, Gokasho, Uji, Kyoto 611-0011, Japan

## Abstract

**Background:**

RNA secondary structure prediction is one major task in bioinformatics, and various computational methods have been proposed so far. Pseudoknot is one of the typical substructures appearing in several RNAs, and plays an important role in some biological processes. Prediction of RNA secondary structure with pseudoknots is still challenging since the problem is NP-hard when arbitrary pseudoknots are taken into consideration.

**Results:**

We introduce a new method of predicting RNA secondary structure with pseudoknots based on integer programming. In our formulation, we aim at minimizing the value of the objective function that reflects free energy of a folding structure of an input RNA sequence. We focus on a practical class of pseudoknots by setting constraints appropriately. Experimental results for a set of real RNA sequences show that our proposed method outperforms several existing methods in sensitivity. Furthermore, for a set of sequences of small length, our approach achieved good performance in both sensitivity and specificity.

**Conclusion:**

Our integer programming-based approach for RNA structure prediction is flexible and extensible.

## Background

**Figure 1:**
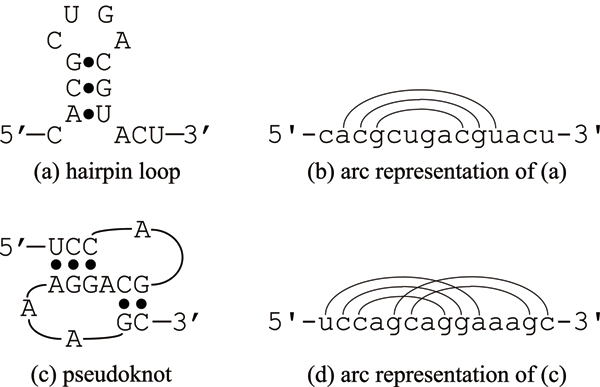
**Example of RNA secondary structure**. (a) Hairpin loop. (b) Arc representation of (a). (c) Pseudoknot. (d) Arc representation of (c).

Functional noncoding RNAs (ncRNAs) have been recognized as regulatory or catalytic molecules, and have received much attention in recent years. It is known that ncRNA genes cover a fair proportion of the whole genome in higher organisms including humans [1], and studying functional ncRNAs is an important task to understand complex mechanism of higher organisms. Structure analysis of ncRNAs will help us elucidate their functions since it is widely recognized that there is correlation between structure and function. Due to difficulty in determining RNA 3D structure (tertiary structure) by experimental techniques, many attempts have so far been made at predicting *secondary structure* given an RNA sequence (primary structure). A secondary structure is defined as a set of hydrogen-bonding base pairs such as Watson-Crick complementary pairs (i.e., A-U and C-G). One of the fundamental secondary structures is shown in Figure 1(a), which is called a hairpin loop or stem loop. Another diagrammatic representation is arc depiction where base pairs are connected by arcs over the RNA sequence (see Figure 1(b)). Among several RNAs, including rRNAs, tmRNAs and viral RNAs, there are substructures called *pseudoknots *(Figure 1(c)) where some arcs over the sequence cross in the arc representation as shown in Figure 1(d). Prediction of RNA secondary structure with pseudoknots has increased in importance since pseudoknots have been known to play an important role in a number of RNA functions such as ribosomal frameshifting and splicing. Furthermore, a biologically reliable database called PseudoBase [2] has been constructed, which contains structural, functional and sequence data on RNA pseudoknots.

Many single-stranded RNAs are considered to fold back on themselves to be thermodynamically stable. This idea has led to the development of several algorithms that minimize the equilibrium free energy of RNA. In the pioneering work, we refer to the Zuker's algorithm [3] that predicts secondary structure with the lowest free energy by using a dynamic programming (DP) technique, and its implementation named Mfold [4] is one of the major softwares for RNA secondary structure prediction. The time complexity of the Zuker's algorithm is *O*(*n*^3^) where *n *is the length of an input RNA sequence. However, this algorithm cannot deal with pseudoknots. To predict pseudoknotted structure, several DP algorithms that minimize free energy in the range from *O*(*n*^4^) to *O*(*n*^6^) time were proposed including PKNOTS [5], pknotsRG [6] and iterated loop matching (ILM) [7]. These algorithms focus on some restricted pseudoknots for solvability in polynomial time since prediction of arbitrary pseudoknotted structure was proven to be NP-hard [8,9]. 

There have also been several grammatical approaches to modeling some kinds of pseudoknots, including use of tree adjoining grammar [10,11], crossed-interaction grammar [12], parallel communicating grammar [13] and multiple context-free grammar [14]. In these grammatical approaches, secondary structure prediction can be interpreted as parsing of the grammars, which can be addressed in *O*(*n*^4^) to *O*(*n*^6^) time. Although performance of these grammar-based methods is comparable to that of energy-based methods, grammatical approaches have the advantage of the capability of constructing profile (or modeling consensus structure) when multiple structural alignment is given in advance.

The sequence alignment information is also useful for RNA secondary structure prediction. Sankoff [15] proposed a DP algorithm that can simultaneously solve the sequence alignment and folding problems for multiple sequences. Later, Sankoff's algorithm was improved by Mathews and Turner [16], where their proposed algorithm is called Dynalign. Dynalign combines free energy minimization and comparative sequence analysis to find a low free energy structure common to two sequences without requiring any sequence identity.

Recently, Parisien and Major [17] presented a new analysis of RNA secondary structure prediction by incorporating all base pairs including noncanonical ones. They introduced a new representation of the nucleotide relationships in structural RNAs, called the nucleotide cyclic motif (NCM). NCM is incorporated into the scoring function of a folding algorithm, which is also based on DP.

Returning to the viewpoint of energy minimization, RNA secondary structure prediction can be regarded as a kind of optimization problem. In fact, several problems in bioinformatics can be formulated as combinatorial optimization problems. One of the successful applications is RAPTOR [18], which calculates protein threading using an *integer programming *(IP) formulation. Although IP problems are known to be NP-hard, descriptive power of IP is strong and flexible. In addition, recent commercial optimization softwares can deal with relatively large-scale instances even if a problem is computationally hard to solve. 

In this paper, we present a new method of predicting RNA secondary structure with pseudoknots based on IP. In our model, thermodynamic information is incorporated into the objective function whose value is to be minimized, and structural information is represented by constraints. It should be noted that we focus on a practical class of pseudoknots by setting constraints appropriately. We use the CPLEX software [19] to solve the IP problem and evaluate our method on a set of RNA sequences contained in PseudoBase [2] and Rfam [20]. Advantages of our proposed method are summarized as follows:

• Our prediction method using IP is flexible. Specifically, various types of secondary structures can be handled by adding or removing constraints. In fact, we can describe pseudoknot-free structures as well as generalized planar pseudoknots (see [8] for definition) by IP formulations, and in this paper we provide a modeling of a certain type of *recursive *pseudoknot (see Methods).

• The IP-based method outperforms three existing methods PKNOTS, pknotsRG and ILM in sensitivity for 34 RNA sequences known to have pseudoknots. In particular, for a set of sequences of small length (≤ 46), our approach achieved good performance in both sensitivity and specificity.

• Once we can model the prediction problem by an IP formulation, we need not develop any algorithm from scratch because high-performance solvers for IP are now available. Thus, we can focus on how to describe the topology of structure and how to assign appropriate scores to the model.

• The IP-based approach is extensible. If we have incomplete data on secondary structure where parts of the structure (i.e., a set of base pairs) have been determined by experiment, we can complement the incomplete structure by modeling the known parts of the structure as constraints and solving the IP problem.

## Methods

### Definitions of RNA secondary structure and pseudoknot

In this section we will describe the preliminary definitions of RNA secondary structure including pseudoknots.

**Definition 1 **(RNA secondary structure). An RNA sequence is represented by a string of *n *characters *s *= *s*_1_*s*_2 _⋯ s_*n *_where *s*_*i *_∈ {*A*, *C*, *G*, *U*}. A *secondary structure *of the sequence *s *is defined as a set *S *of base pairs (*s*_*i*_, *s*_*j*_) such that the following conditions are satisfied:

1. 1 ≤ *i *<*j *≤ *n*, meaning, two bases that form a pair must be located at different positions.

2. *j *- *i *> *t *where *t *is a small positive constant, meaning, the sequence does not fold too sharply on itself.

3. For all base pairs (*s*_*i*_, *s*_*j*_) and (*s*_*i*_', *s*_*j*_') in *S*, *i *= *i*' if and only if *j *= *j*', meaning, each base can be paired with at most one base.

Here, we allow only Watson-Crick base pairs (*A*, *U*) and (*C*, *G*), and a wobble base pair (*G*, *U*) to form the secondary structure, which we call *valid *base pairs.

**Definition 2 **(Pseudoknot). An RNA secondary structure *S *is said to contain a *pseudoknot *if and only if there exist (*s*_*i*_, *s*_*j*_), (*s*'_*i*_, *s*'_*j*_) ∈ *S *(*i *<*i*') such that *i *<*i*' <*j *<*j*'.

**Definition 3 **(Pseudoknot free). An RNA secondary structure *S *is called *pseudoknot free *if and only if for all pairs (*s*_*i*_, *s*_*j*_), (*s*_*i*_', *s*_*j*_') ∈ *S *(*i *<*i*'), one of the following conditions is satisfied:

1. *i *<*j *<*i*' <*j*', i.e., (*s*_*i*_, *s*_*j*_) precedes (*s*_*i'*_, *s*_*j'*_), or

2. *i *<*i*' <*j*' <*j*, i.e., (*s*_*i*_, *s*_*j*_) includes (*s*_*i'*_, *s*_*j'*_).

There are various kinds of pseudoknots, depending on how arcs representing base pairs cross above the sequence. For tractability in computational complexity, several classes of pseudoknots were proposed.

**Figure 2:**
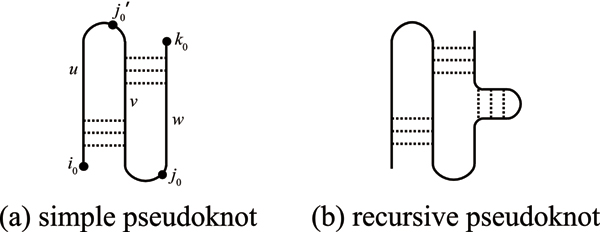
**Illustration of pseudoknots**. (a) Simple pseudoknot. (b) Recursive pseudoknot.

**Definition 4 **(Simple pseudoknot [8]). Let $s_{i_{0}}s_{i_{0}+1}\cdots s_{k_{0}}$ be a consecutive RNA subsequence. A set of base pairs ${S^{\prime }}_{i_{0},k_{0}}$ is called a *simple pseudoknot *if there exist positions *j*_0_, ${j^{\prime }}_{0}$ (*i*_0 _<${j^{\prime }}_{0}$ <*j*_0 _<*k*_0_) satisfying the following conditions (see Figure 2(a)):

1. Each (*i*, *j*) ∈ ${S^{\prime }}_{i_{0},k_{0}}$ satisfies either *i*_0 _≤ *i *<${j^{\prime }}_{0}$ ≤ *j *<*j*_0 _or ${j^{\prime }}_{0}$ ≤ *i *<*j*_0 _≤ *j *≤ *k*_0_.

2. There exists (*i*, *j*) ∈ ${S^{\prime }}_{i_{0},k_{0}}$ satisfying *i*_0 _≤ *i *<${j^{\prime }}_{0}$ ≤ *j *<*j*_0_.

3. There exists (*i*, *j*) ∈ ${S^{\prime }}_{i_{0},k_{0}}$ satisfying ${j^{\prime }}_{0}$ ≤ *i *<*j*_0 _≤ *j *≤ *k*_0_.

4. If pairs (*i*, *j*) and (*i*', *j*') in ${S^{\prime }}_{i_{0},k_{0}}$ satisfy either *i *<*i*' <${j^{\prime }}_{0}$ or ${j^{\prime }}_{0}$ ≤ *i *<* i*', then *j *> *j*' holds.

**Definition 5 **(Recursive pseudoknot [8]). If internal unpaired regions of a simple pseudoknot (e.g., subsequences *u*, *v *and *w *in Figure 2(a)) fold into pseudoknot-free structure and/or simple pseudoknot, the structure is called a *recursive pseudoknot *(see Figure 2(b)).

In this paper, we propose an integer programming-based method that can predict a subclass of recursive pseudoknots. It should be noted that our formulation allows unpaired regions of a simple pseudoknot to fold into pseudoknot-free structure only.

### Integer programming-based model

#### Definitions of integer programming

Integer programming (IP) is an extension of linear programming. A linear programming (LP) problem is one of the optimization problems, which optimizes a linear function subject to linear equality and/or inequality constraints. An LP problem is composed of *decision variables *whose values are to be decided in some optimal fashion, an *objective function *to be maximized or minimized, and a set of *constraints*. The constraints consist of linear equalities and/or inequalities with respect to the decision variables. When the decision variables are required to be integer, the problem is called an *integer programming (IP) problem*. In general, an IP problem can be written as follows:

$$\begin{array}{ll} \text{minimize} & \sum_{j=1}^{n}c_{j}x_{j}\\ \text{subject to} & \begin{array}{cc} \sum_{j=1}^{n}\alpha_{ij}x_{j}\leq b_{i}, & i=1,2,...,m \end{array}\\ & \begin{array}{cc} x_{j}\in {\mathbb{Z}}^{+}, & j=1,2,...,n \end{array} \end{array}$$

where *a*_*ij*_, *b*_*i*_, *c*_*j *_∈ ℝ (*i *= 1, 2, ..., *m*; *j *= 1, 2, ..., *n*) and *x*_*j *_(*j *= 1, 2, ..., *n*) denotes a decision variable defined over a set of nonnegative integers ℤ^+^. Note that the maximization problem is equivalent to the minimization problem where the sign of the objective function is inverted.

#### Formulation for RNA pseudoknot prediction

We will formulate the RNA pseudoknotted structure prediction problem as an IP problem. Specifically, we define the decision variables, the objective function and the linear constraints with respect to the decision variables.

**Figure 3:**
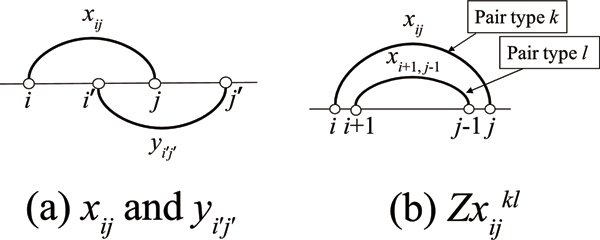
**Illustration of decision variables**. (a) *x*_*ij *_and *y*_*i' j'*_. (b) $z_{x_{ij}}^{kl}$.

We first define the following decision variables:

$$x_{ij},y_{ij}=\{\begin{array}{ll} 1 & ((s_{i},s_{j})\text{ is a valid pair}),\\ 0 & \left(\text{otherwise}\right) \end{array}$$

for *i*, *j *= 1, 2, ..., *n*. The difference between *x*_*ij *_and *y*_*ij *_is that *x*_*ij *_= 1 corresponds to an arc that connects two bases drawn above the sequence, while *y*_*ij *_= 1 represents an arc drawn below the sequence (see Figure 3(a)).

In order to incorporate stacking energy into decision variables, we use 6 × 6 (row × column, resp.) energy parameter matrix E = (*e*_*kl*_) (*k*, *l *= 1, 2, ..., 6) shown in Table 1 . This matrix provides stacking energy parameters for RNA folding at 37°C given by Mfold version 3.0 [4]. Note that the value of *k *denotes the "type" of six valid pairs. For example, *k *= 1 indicates A-U pair and we call it "type 1." This statement also holds for the value of *l*.

**Table 1:** Stacking energy parameter matrix *E *[4].

	A-U	C-G	G-C	G-U	U-G	U-A
A-U	-1.1	-2.1	-2.2	-1.4	-0.9	-0.6
C-G	-2.1	-2.4	-3.3	-2.1	-2.1	-1.4
G-C	-2.2	-3.3	-3.4	-2.5	-2.4	-1.5
G-U	-1.4	-2.1	-2.5	-1.3	-1.3	-0.5
U-G	-0.9	-2.1	-2.4	-1.3	-1.3	-1.0
U-A	-0.6	-1.4	-1.5	-0.5	-1.0	-0.3

We then define variables for representing the stacking pair of (*s*_*i*_, *s*_*j*_) and (*s*_*i*+1_, *s*_*j*-1_) as follows:

$$\begin{array}{c} z_{x_{ij}}^{kl}=\{\begin{array}{ll} 1 & (x_{ij}=1\text{ with type }k\text{ and }x_{i+1,j-1}=1\text{ with type }l),\\ 0 & (\text{otherwise}), \end{array}\\ z_{y_{ij}}^{kl}=\{\begin{array}{ll} 1 & (y_{ij}=1\text{ with type }k\text{ and }y_{i+1,j-1}=1\text{ with type }l),\\ 0 & \left(\text{otherwise}\right) \end{array} \end{array}$$

for *i*, *j *= 1, 2, ..., *n *and *k*, *l *= 1, 2, ..., 6 (see Figure 3(b)). Note that the above definitions include illegal variables *x*_*i*+1,0 _and *x*_*n*+1,*j*-1 _for each *i *and *j *respectively (which also applies to *y*_*ij*_), though we allow this notation for simplicity.

Let $L_{x_{i}}$ = 1 and $R_{x_{i}}$ = 1 if and only if the base *s*_*i *_pairs with some base at any other position greater than *i *and less than *i *respectively. In other words, $L_{x_{i}}$ = 1 means that *s*_*i *_is on the left side of a base pair and $R_{x_{i}}$ = 1 means that *s*_*i *_is on the right side of a base pair. This set of variables also applies to *y*_*ij*_, defined by $L_{y_{i}}$ and $R_{y_{i}}$.

**Figure 4:**
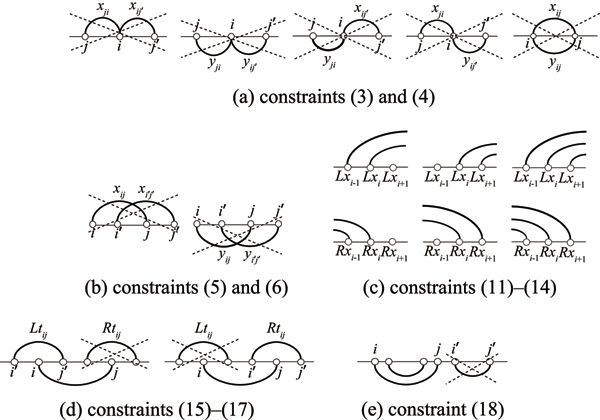
**Illustration of several constraints**. (a) Constraints (3) and (4). (b) Constraints (5) and (6). (c) Constraints (11)–(14). (d) Constraints (15)–(17). (e) Constraint (18).

We will use variables $L_{t_{ij}}$ and $R_{t_{ij}}$ to represent a recursive pseudoknotted structure. We let $L_{t_{ij}}$ = 1 if and only if for a base pair (*s*_*i*_, *s*_*j*_) below the sequence, there is at least one base pair above the sequence (*s*_*i'*_, *s*_*j'*_) such that *i*' <*i *<*j*'. Similarly, let $R_{t_{ij}}$ = 1 if and only if for a pair (*s*_*i*_, *s*_*j*_) below the sequence, there is at least one base pair above the sequence (*s*_*i'*_, *s*_*j'*_) such that *i*' <*j *<*j*' (see Figure 4(d)). With these variables, we can formulate an IP problem for RNA pseudoknot prediction as follows:

$$\begin{array}{cc} \text{minimize} & \sum_{i=1}^{n}\sum_{j=1}^{n}\sum_{k=1}^{6}\sum_{l=1}^{6}\left(e_{kl}z_{x_{ij}}^{kl}+\sigma e_{kl}z_{y_{ij}}^{kl}\right) \end{array}$$

subject to

(1) $$z_{x_{ij}}^{kl}\leq \frac{x_{ij}+x_{i+1,j-1}}{2}$$

((*s_i_,s_j_*) is type *k* and (*s*_*i*+1_,*s*_*j*-1_) is type *l*),

(2) $$z_{y_{ij}}^{kl}\leq \frac{y_{ij}+y_{i+1,j-1}}{2}$$

((*s_i_,s_j_*) is type *k* and (*s*_*i*+1_,*s*_*j*-1_) is type *l*),

(3) $$\sum_{j=1}^{i-1}x_{ji}+\sum_{j^{\prime }=i+1}^{n}x_{ij^{\prime }}+\sum_{j=1}^{i-1}y_{ji}+\sum_{j^{\prime }=i+1}^{n}y_{ij^{\prime }}\leq 1,$$

(4) *x*_*ij *_+ *y*_*ij *_≤ 1,

(5) *x*_*ij *_+ *x*_*i*'*j*' _≤ 1 (∀*i *<*i*' <*j *<*j*'),

(6) *y*_*ij *_+ *y*_*i*'*j*' _≤ 1 (∀*i *<*i*' <*j *<*j*'),

(7) $$L_{x_{i}}-\sum_{j=i+1}^{n}x_{ij}=0,$$

(8) $$R_{x_{i}}-\sum_{j=1}^{i-1}x_{ji}=0,$$

(9) $$L_{y_{i}}-\sum_{j=i+1}^{n}y_{ij}=0,$$

(10) $$R_{y_{i}}-\sum_{j=1}^{i-1}y_{ji}=0,$$

(11) $$L_{x_{i-1}}+(1-L_{x_{i}})+L_{x_{i+1}}\geq 1,$$

(12) $$R_{x_{i-1}}+(1-R_{x_{i}})+R_{x_{i+1}}\geq 1,$$

(13) $$L_{y_{i-1}}+(1-L_{y_{i}})+L_{y_{i+1}}\geq 1,$$

(14) $$R_{y_{i-1}}+(1-R_{y_{i}})+R_{y_{i+1}}\geq 1,$$

(15) $$L_{t_{ij}}\geq \frac{1}{n^{2}}\sum_{i^{\prime }<i<j^{\prime }<j}x_{i^{\prime }j^{\prime }},$$

(16) $$R_{t_{ij}}\geq \frac{1}{n^{2}}\sum_{i<i^{\prime }<j<j^{\prime }}x_{i^{\prime }j^{\prime }},$$

(17) $$L_{t_{ij}}+R_{t_{ij}}\leq 1,$$

(18) *y*_*ij *_+ *y*_*i*'*j*' _≤ 1 (∀*i *<*j *<*i*' <*j*'),

(19) $$x_{ij},y_{ij},z_{x_{ij}}^{kl},z_{y_{ij}}^{kl},L_{x_{i}},L_{y_{i}},R_{x_{i}},R_{y_{i}},L_{t_{ij}},R_{t_{ij}}\in \{0,1\}$$

where *i*, *j*, *i*', *j*' = 1, 2, ..., *n*; *k*, *l *= 1, 2, ..., 6.

The first term in summations of the objective function is the sum of the overall energy of stacking base pairs formed above the sequence, while the second term is the sum of the overall energy of the stacking base pairs formed below the sequence. We multiply the second term by a positive weighting parameter *σ*. The weighting parameter restricts the occurrence of base pairs below the sequence. This is to take into account that pseudoknotted structure frequently does not appear in nature. The value of *σ *suggested in [5] is *σ *< 1, though the early work employed a dynamic programming-based approach. In our model, we define *σ *∈ {0.55, 0.6, 0.65, 0.7, 0.75, 0.8}.

Constraints (1) and (2) mean that if (*s*_*i*_, *s*_*j*_) is a valid pair with type *k *and (*s*_*i*+1_, *s*_*j*-1_) is a valid pair with type *l*, the energy parameter associated with (*k*, *l*) stacking type will contribute to the total energy of the structure (Figure 3(b)). Constraints (3) and (4) say that each base can be paired with at most one base regardless of whether the pair is formed above or below the sequence (Figure 4(a)). Constraints (5) and (6) inhibit crossing pairs both above the sequence and below the sequence (Figure 4(b)). In constraints (7), (8), (9) and (10), $L_{x_{i}}$, $R_{x_{i}}$, $L_{y_{i}}$ and $R_{y_{i}}$ are defined respectively. Constraints (11), (12), (13) and (14) guarantee that if a base is paired with another one, its adjacent base must also form a base pair (Figure 4(c)). The purpose of these constraints is to promote stacking pairs because they are known to help to stabilize the structure. Constraints (15) and (16) define $L_{t_{ij}}$ and $R_{t_{ij}}$ respectively. In the constraint (17), if (*s*_*i*_, *s*_*j*_) is a base pair formed below the sequence, either $L_{t_{ij}}$ or $R_{t_{ij}}$ can take the value 1. This means that the crossing region can occur once at a time (Figure 4(d)). Notice that base pairs can be formed recursively in the loop regions, for example, the region from *i*' to *i *and the region from *i *to *j*' in Figure 4(d). By virtue of constraints (15), (16), (17), this IP model can handle a subclass of recursive pseudoknots where internal unpaired regions of a simple pseudoknot can have pseudoknot-free structure. The constraint (18) is used for prohibiting bifurcation structure from occurring below the sequence (Figure 4(e)). The constraint (19) guarantees all variables to be either 0 or 1.

## Results and discussion

### Data set

Test sequences that are known to form pseudoknots were taken from PseudoBase [2], where we selected 34 sequences of several kinds of secondary structures and of various lengths (21–137 bases) from different families (viral 3'UTR, mRNA, rRNA, ribozymes and tRNA-like). Specifically, we first selected a set of sequences of lengths 20–140 bases uniformly. We then checked the secondary structure of each sequence and removed sequences of similar secondary structure from the set.

Other test sequences that do not contain any pseudoknots were obtained from Rfam [20]. Seven pseudoknot-free sequences from different families were selected in the same way as the sequences that contain pseudoknots, so that their secondary structures and lengths are different (26–88 bases).

### Implementation

After formulating the IP problem for prediction of RNA secondary structure, we employed the optimizer software called ILOG CPLEX 10.1 [19] to solve the IP formulation. CPLEX is a commercial software that can solve mathematical optimization problems, including IP problems. We implemented the IP formulation by C++ and included the C++ optimization library provided by CPLEX on a machine with Intel Xeon CPU 5160 3.00 GHz and 8.00 GB RAM.

In our implementation, we reduced the set of variables *x*_*ij *_and *y*_*ij *_to solve the problem fast. During implementation of *x*_*ij *_and *y*_*ij*_, we focused on the fact that at least two base pairs are likely to appear consecutively on the sequence. We considered not only the pair of (*s*_*i*_, *s*_*j*_) but also its adjacent pairs, i.e., (*s*_*i*-1_, *s*_*j*+1_) and (*s*_*i*+1_, *s*_*j*-1_). If (*s*_*i*_, *s*_*j*_) and at least one of those adjacent pairs are valid, we implemented *x*_*ij *_and *y*_*ij *_as decision variables.

### Tests

Prediction accuracy of our method was measured by the sensitivity and specificity. Specificity is defined as the proportion of the number of correctly predicted base pairs to the number of base pairs of the known structure. Specificity is defined as the proportion of the number of correctly predicted base pairs to the total number of base pairs predicted by the algorithm.

We carried out pseudoknotted structure prediction with each value of *σ *in order to determine the most proper value (see Table 2 . We then chose *σ *that yielded the highest average prediction accuracy on our data set. From Table 2 , the best value of *σ *is 0.70. However, increasing or decreasing value of *σ *did not show any definitive way to find the optimal value of *σ*.

**Table 2:** Average sensitivity and specificity of each value of *σ*.

*σ*	Avg. sensitivity (%)	Avg. specificity (%)
0.55	75.67	64.92
0.60	72.70	62.21
0.65	70.53	60.86
**0.70**	75.91	65.40
0.75	75.09	65.02
0.80	70.53	60.89

We compared the prediction results on the best *σ *(*σ *= 0.70) of IP with the results from ILM [7], pknotsRG [6] and PKNOTS [5], shown in Table 3 . Note that PKB number is an identification number of each RNA sequence used in PseudoBase. Moreover, we compared computation time of each method (see Table 4 ). As the table shows, computation time of the IP-based method depends on the sequence length. Specifically, as the sequence length elongates, the computation time increases exponentially.

**Table 3:** Prediction results of the IP-based method (*σ *= 0.7) and prediction results of the other algorithms.

PKB num.	Length	Sensitivity (%)				Specificity (%)			
		
		**IP**	ILM	pknotsRG	PKNOTS	**IP**	ILM	pknotsRG	PKNOTS
PKB115	21	**100.00**	66.67	**100.00**	66.67	**100.00**	**100.00**	**100.00**	**100.00**
PKB102	24	**100.00**	87.50	**100.00**	87.50	80.00	77.78	**88.89**	77.78
PKB119	24	**88.89**	0.00	77.78	66.67	88.89	0.00	87.50	**100.00**
PKB103	25	**100.00**	57.14	57.14	42.86	**70.00**	66.67	50.00	50.00
PKB123	26	**90.00**	**90.00**	70.00	60.00	**90.00**	**90.00**	77.78	85.71
PKB154	26	**100.00**	90.00	**100.00**	**100.00**	**100.00**	**100.00**	**100.00**	**100.00**
PKB152	26	**100.00**	90.00	**100.00**	**100.00**	**90.91**	90.00	**90.91**	**90.91**
PKB126	27	**100.00**	**100.00**	**100.00**	**100.00**	**100.00**	**100.00**	**100.00**	**100.00**
PKB124	29	**100.00**	70.00	**100.00**	70.00	**100.00**	**100.00**	**100.00**	**100.00**
PKB100	31	**100.00**	91.67	91.67	**100.00**	92.31	91.67	91.67	**100.00**
PKB105	32	**88.89**	**88.89**	**88.89**	**88.89**	88.89	**100.00**	**100.00**	**100.00**
PKB118	33	**90.00**	80.00	**90.00**	80.00	**81.82**	72.73	**81.82**	72.73
PKB120	36	**100.00**	**100.00**	**100.00**	**100.00**	85.71	**100.00**	**100.00**	**100.00**
PKB65	46	**93.33**	**93.33**	0.00	73.33	70.00	**87.50**	0.00	68.75
PKB205	48	**23.08**	0.00	**23.08**	**23.08**	15.00	0.00	**23.08**	**23.08**
PKB147	51	**77.78**	55.56	50.00	50.00	**63.64**	55.56	47.37	52.94
PKB248	66	**80.00**	65.00	35.00	65.00	64.00	**72.22**	33.33	61.90
PKB72	67	**100.00**	0.00	35.29	**100.00**	62.96	0.00	31.58	**73.91**
PKB140	69	**73.91**	65.22	13.04	43.48	62.96	**65.22**	15.79	50.00
PKB143	71	62.50	**91.67**	29.17	70.83	48.39	**78.57**	29.17	68.00
PKB144	71	79.17	**95.83**	29.17	66.67	70.37	**95.83**	31.82	72.73
PKB173	73	63.64	**77.27**	36.36	**77.27**	50.00	68.00	44.44	**77.27**
PKB276	73	42.86	**76.19**	33.33	38.10	30.00	**80.00**	30.43	38.10
PKB275	85	65.38	**96.15**	15.38	50.00	50.00	**83.33**	15.38	72.22
PKB75	88	71.88	**93.75**	18.75	81.25	62.16	**85.71**	22.22	81.25
PKB76	89	56.00	**100.00**	52.00	44.00	40.00	**69.44**	48.15	34.38
PKB164	96	38.71	**74.19**	48.39	35.48	29.27	**65.71**	53.57	34.38
PKB168	105	61.76	**79.41**	35.29	76.47	50.00	81.82	34.29	**86.67**
PKB252	110	15.38	**97.44**	35.90	**97.44**	13.04	80.85	35.90	**92.68**
PKB191	113	74.36	**82.05**	56.41	71.79	61.70	**80.00**	55.00	73.68
PKB135	116	82.05	**84.62**	38.46	74.36	69.57	**84.62**	40.54	74.36
PKB236	120	50.00	37.50	0.00	**67.50**	42.55	30.61	0.00	**72.97**
PKB137	133	70.45	84.09	63.64	**86.36**	65.96	80.43	63.64	**86.36**
PKB134	137	40.91	59.09	40.91	**84.09**	33.33	50.00	41.86	**80.43**

Average		**75.91**	74.12	54.85	71.74	65.40	73.07	54.89	**75.09**

**Table 4:** Computation time of the IP-based method (*σ *= 0.7) and the other algorithms. Note that it is difficult to measure the exact computation time of pknotsRG since it is provided as an interactive system, and the time listed below is a rough estimate when running pknotsRG.

Seq. length	Avg. computation time (sec.)			
	
	**IP**	ILM	pknotsRG	PKNOTS
21–46	0.14	0.05	<1.00	0.73
48–66	16.40	0.15	<1.00	24.67
67–137	6641.53	0.31	<1.00	1205.61

We also tested the IP-based model with the pseudoknot-free sequences. The IP-based model with *σ *= 0.7 gives 63.30% of average sensitivity and 47.89% of average specificity. Since the input data set is a set of pseudoknot-free sequences, the appropriate value of *σ *should be close to 0. Hence, we tested the model with *σ *∈ {0.05, 0.1, 0.15, 0.2}. The results showed that *σ *= 0.1 gives the best average sensitivity (72.18%) and the best average specificity (55.93%).

## Discussion

We averaged the sensitivity and specificity to examine the overall performance of each prediction method. Our IP-based method gives 75.91% sensitivity, which is the highest of the four models. For specificity, the IP-based method gives 65.40%, which is the third highest, and the best specificity is given by PKNOTS (75.09%). From Table 3 , it is obvious that for short sequences (less than 70 bases), the accuracy of IP is very high and at least comparable to the other algorithms. For sequences of lengths 70–116 bases, ILM yields the highest sensitivity and specificity for most of the sequences in this group. One reason might be that some statistical information that ILM uses plays a key role in achieving good accuracy for long sequences. It is a challenge to incorporate some kind of statistical information into our IP-based method. 

The reason why the IP-based method does not yield high specificity could be that when IP is being optimized, *x*_*ij *_and *y*_*ij *_are assigned to be 1 as many as possible because the energy parameters are negative values and the objective function is to minimize the overall energy. This results in the occurrence of a number of false positive pairs. Therefore, the specificity of our method is lower than that of the other methods.

**Figure 5:**
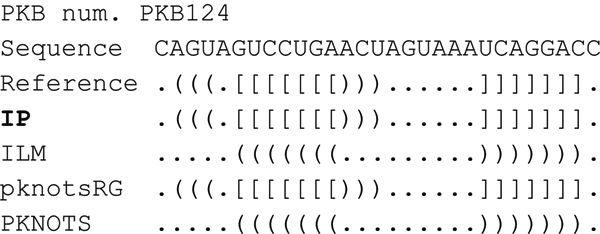
Comparison of predicted structure for PKB124 between the four algorithms.

When we observed predicted structures, we found that IP always outputs pseudoknotted structure. Although ILM and PKNOTS provide good specificity, it is not guaranteed that base pairs forming pseudoknots are always predicted, especially for short sequences (see Figure 5). Since the proportion of base pairs that constitute pseudoknots in RNA secondary structure is small compared to the total number of base pairs, the specificity of those algorithms is high.

We considered using the leave-one-out strategy to verify the optimality of *σ*, resulting in a drop in accuracy (70.19% average sensitivity and 60.33% average specificity). Among 34 leave-one-out experiments, there are 28 experiments where *σ *= 0.70 yields the best prediction result. However, there is one experiment that gives low accuracy (11.11% sensitivity and 8.70% specificity), where PKB147 is used as a test sequence and *σ *= 0.75. As a result, the average sensitivity and specificity is lower than the results shown in Table 3  where we do not perform the cross validation for *σ*. As stated before, *σ *is a fixed weighting parameter to restrict the occurrence of base pairs that form pseudoknots. It is not necessary to train the value of *σ *because our main purpose is not to optimize the *σ *parameter but to test the applicability of the IP-based model to the secondary structure prediction problem. If we aim at training parameters of the prediction algorithm, we should consider not only *σ *but also the other coefficients of the objective function (i.e., energy parameters), which is left as future work.

As explained in Implementation subsection, we implemented decision variables *x*_*ij *_and *y*_*ij *_based on two valid consecutive base pairs. It should be noted that the number of these variables is fewer than the number of original variables before the reduction (seeTable 5 ). Such a reduction contributes to lower memory usage, which leads to the capability of dealing with long sequences for prediction. Furthermore, we also considered three valid consecutive pairs, i.e., (*s*_*i*-2_, *s*_*j*+2_), (*s*_*i*-1_, *s*_*j*+1_), (*s*_*i*+1_, *s*_*j*-1_), and (*s*_*i*+2_, *s*_*j*-2_) for each pair of (*s*_*i*_, *s*_*j*_) so that we can further reduce the number of variables and can expect to increase the prediction accuracy. However, the performance using two consecutive pairs was better than using three consecutive pairs. This might reveal that reducing too many variables could make the problem harder to optimize, which results in worse prediction accuracy and more computation time. Note that in this paper we only show the results using variables on two consecutive pairs.

**Table 5:** Number of variables before and after implementing variable reduction.

Seq. length	21	137
Before reduction	12244	400984
After reduction	2938	117240

Since actual RNA structure does not necessarily have the lowest energy, it is important to consider suboptimal structures, which might improve prediction accuracy. From this viewpoint, Zuker [21] proposed an efficient suboptimal folding algorithm. In our case, the optimal solution depends on how the solver (CPLEX) works out the IP problem, which is hidden to us, and thus we cannot retrieve suboptimal solutions from the solver. However, once an optimal solution (structure) is determined by the solver, we might be able to calculate some of the suboptimal structures by describing constraints where some stacking pairs are forbidden.

## Conclusion

We proposed an integer programming (IP)-based method of predicting RNA secondary structure with a certain kind of recursive pseudoknot. Prediction tests on a set of RNA sequences were carried out, which showed good performance in accuracy for a data set of relatively small length. Furthermore, our method achieved higher average sensitivity than that of several existing prediction methods for the same test set, as well as guaranteed that base pairs involved in pseudoknots are always predicted.

We also tested the IP-based model with pseudoknot-free sequences. Although this IP formulation is specifically designed for pseudoknotted structure, the results showed that the IP-based method can also be useful in predicting secondary structure in the absence of pseudoknots. However, based on this IP formulation, we can formulate another IP-based model to predict pseudoknot-free structure and would be able to obtain better prediction accuracy.

As described before, our IP-based approach is flexible and extensible. Recall that the IP-based prediction method takes much time for long RNA sequences. In general, computation time of IP is exponential to the number of variables and thus it is important to reduce the number of variables. Although in this paper we used an IP variable (e.g., *x*_*ij*_) to represent just one base pair, we would be able to define variables in such a way that they can handle larger units at a time, which results in further reduction of the number of variables. A kind of divide-and-conquer approach could also be useful where a long input sequence is divided into several subsequences of short length and then we apply the IP-based prediction method to each short subsequence. This approach will shorten the computation time, as well as increase the prediction accuracy, which is left as our future work.

## Competing interests

The authors declare that they have no competing interests.

## Authors' contributions

UP carried out all experiments and drafted the manuscript. YK participated in the design of the integer programming formulation and drafted the manuscript. TA conceived the idea of the work and helped to draft the manuscript. All authors read and approved the final manuscript.

## References

[B1] Shabalina SA, Spiridonov NA (2004). The mammalian transcriptome and the function of non-coding DNA sequences. Genome Biology.

[B2] Batenburg FHDvan, Gultyaev AP, Pleij CWA, Ng J, Oliehoek J (2000). PseudoBase: a database with RNA pseudoknots. Nucleic Acids Research.

[B3] Zuker M, Stiegler P (1981). Optimal computer folding of large RNA sequences using thermodynamics and auxiliary information. Nucleic Acids Research.

[B4] Zuker M (2003). Mfold web server for nucleic acid folding and hybridization prediction. Nucleic Acids Research.

[B5] Rivas E, Eddy SR (1999). A dynamic programming algorithm for RNA structure prediction including pseudoknots. Journal of Molecular Biology.

[B6] Reeder J, Giegerich R (2004). Design, implementation and evaluation of a practical pseudoknot folding algorithm based on thermodynamics. BMC Bioinformatics.

[B7] Ruan J, Stormo GD, Zhang W (2004). An iterated loop matching approach to the prediction of RNA secondary structures with pseudoknots. Bioinformatics.

[B8] Akutsu T (2000). Dynamic programming algorithms for RNA secondary structure prediction with pseudoknots. Discrete Applied Mathematics.

[B9] Lyngsø RB, Pedersen CNS (2000). RNA pseudoknot prediction in energy-based models. Journal of Computational Biology.

[B10] Uemura Y, Hasegawa A, Kobayashi S, Yokomori T (1999). Tree adjoining grammars for RNA structure prediction. Theoretical Computer Science.

[B11] Matsui H, Sato K, Sakakibara Y (2005). Pair stochastic tree adjoining grammars for aligning and predicting pseudoknot RNA structures. Bioinformatics.

[B12] Rivas E, Eddy SR (2000). The language of RNA: a formal grammar that includes pseudoknots. Bioinformatics.

[B13] Cai L, Malmberg RL, Wu Y (2003). Stochastic modeling of RNA pseudoknotted structures: a grammatical approach. Bioinformatics.

[B14] Kato Y, Seki H, Kasami T (2006). RNA pseudoknotted structure prediction using stochastic multiple context-free grammar. IPSJ Transactions on Bioinformatics.

[B15] Sankoff D (1985). Simultaneous solution of the RNA folding, alignment and protosequence problems. SIAM Journal on Applied Mathematics.

[B16] Mathews DH, Turner DH (2002). Dynalign: an algorithm for finding the secondary structure common to two RNA sequences. Journal of Molecular Biology.

[B17] Parisien M, Major F (2008). The MC-Fold and MC-Sym pipeline infers RNA structure from sequence data. Nature.

[B18] Xu J, Li M, Kim D, Xu Y (2003). RAPTOR: optimal protein threading by linear programming. J Bioinform Comput Biol.

[B19] ILOG CPLEX. http://www.ilog.com/products/cplex/.

[B20] Griffiths-Jones S, Moxon S, Marshall M, Khanna A, Eddy SR, Bateman A (2005). Rfam: annotating non-coding RNAs in complete genomes. Nucleic Acids Res.

[B21] Zuker M (1989). On finding all suboptimal foldings of an RNA molecule. Science.

